# The role of sphingosine-1-phosphate in bone remodeling and osteoporosis

**DOI:** 10.1038/s41413-022-00205-0

**Published:** 2022-04-08

**Authors:** Justus M. Grewe, Paul-Richard Knapstein, Antonia Donat, Shan Jiang, Daniel J. Smit, Weixin Xie, Johannes Keller

**Affiliations:** 1 Department of Trauma and Orthopedic Surgery, University Medical Center Hamburg-Eppendorf, 20246 Hamburg, Germany; 2 Clinic and Polyclinic for Vascular Medicine, University Heart Center Hamburg-Eppendorf, 20246 Hamburg, Germany; 3 Institute of Biochemistry and Signal Transduction, University Medical Center Hamburg-Eppendorf, 20246 Hamburg, Germany

**Keywords:** Osteoporosis, Bone

## Abstract

Osteoporosis is a systemic bone disease that affects more than 200 million people worldwide and is caused by the disruption of the equilibrium between osteoclastic bone resorption and osteoblastic bone formation. Sphingosine-1-phosphate (S1P) is a natural, bioactive sphingolipid that has been shown to play a major role in cardiovascular and immunological pathologies by regulating biological and cellular processes, including migration, differentiation, proliferation and survival. Recent studies also suggest a central role for S1P in bone diseases, including osteoporosis; however, the effects of S1P, particularly in bone metabolism, remain to be further elucidated. In this review, we summarize the available literature on the role of S1P in bone metabolism with a focus on osteoporosis. On the cellular level, S1P acts as an osteoclast-osteoblast coupling factor to promote osteoblast proliferation and bone formation. Moreover, the recruitment of osteoclast precursors to resorption sites is regulated by the interplay of S1P gradients and S1P receptor expression. From a clinical perspective, increasing evidence suggests that systemically elevated S1P blood levels may serve as an independent risk factor for osteoporosis-related fractures. Taken together, S1P signaling is a potential therapeutic target and may serve as a novel biomarker in patients with systemic bone disease.

## Introduction

Osteoporosis, a skeletal disorder characterized by low bone mass and microarchitectural deterioration of bone tissue with a consequent increase in bone fragility and fracture risk, is a major health problem.^1^ More than 200 million people are affected worldwide, and osteoporosis is the most prevalent bone-associated disease.^2^ Clinically, this disease often remains undiagnosed until osteoporosis-related fractures (OFs) occur. The lifetime risk of OFs in women and men ranges from 40%–50% and 13%–22%, respectively, and patients suffering from OFs show impaired health-related quality of life, significantly higher mortality rates within the first year and cause high health expenditures.^3–5^

On the cellular level, dysregulated bone remodeling plays a crucial role in the pathogenesis of osteoporosis. Bone remodeling relies on the complex interplay between osteoblasts (OBs), osteoclasts (OCs) and osteocytes and is performed within a temporary anatomic structure termed the basic multicellular unit (BMU).^6^ Bone-resorbing OCs develop from hematopoietic progenitors, while bone-forming OBs are derived from mesenchymal stem cells and can differentiate into osteocytes after being embedded in the bone matrix.^7^ Dysregulation of the balance between OB and OC activity, either through insufficient activity of OBs or excessive activation of OCs, leads to a reduction in bone mass and quality, thereby increasing the risk of bone fractures.

## Sphingosine-1-phosphate

While sphingolipids were previously considered to be structural molecules in cell membranes, they are currently known to serve as bioactive lipids. These bioactive lipids, including sphingosine-1-phosphate (S1P), play major roles in the pathogenesis of various diseases, such as multiple sclerosis, cancer, atherosclerosis, diabetes, and osteoporosis.^8–12^

**Fig. 1:**
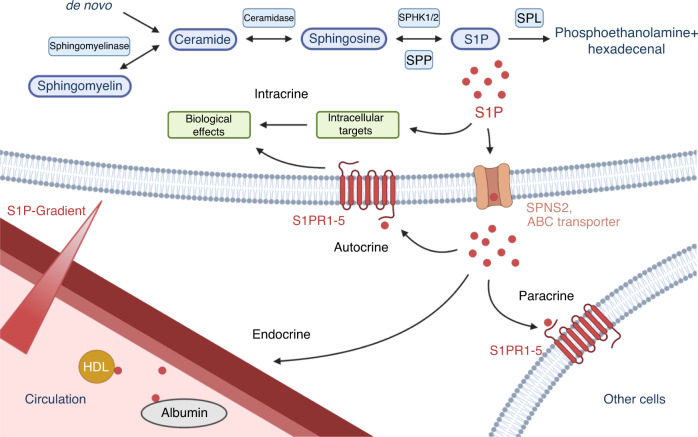
Biosynthesis and degradation of sphingosine-1-phosphate and its intracrine, autocrine, paracrine and endocrine signaling. Ceramides are synthesized de novo or by the cleavage of sphingomyelin. Then, cytosolic ceramidase catalyzes the hydrolysis to sphingosine. Sphingosine kinases 1 and 2 (SPHK1/2) catalyze the phosphorylation of sphingosine to its bioactive metabolite, namely, sphingosine-1-phosphate (S1P). The intracellular S1P concentration is tightly regulated by its degradation via S1P phosphatases (SPPs) and S1P lyases (SPLs). Bioactive S1P molecules are released by the ATP-independent Spinster 2 (SPNS2) transporter or the ATP-dependent ABC transporter. Subsequently, S1P may act on the host cell in an intracrine or autocrine manner, in a paracrine manner on neighboring cells, or in an endocrine manner via the blood circulation

The biosynthesis of S1P begins with the precursor substrate sphingomyelin, which is converted to ceramide by sphingomyelinase through the cleavage of a phosphocholine residue (Fig. 1). In addition, ceramides can also be formed de novo by the condensation of serine and palmitoyl-CoA. Sphingosine (SPH) is cleaved through the subsequent hydrolysis of ceramide by ceramidase and is further phosphorylated by one of two sphingosine kinases (SPHK1 and SPHK2) to generate S1P.^13^ The balance between intracellular concentrations of the proapoptotic precursors sphingosine and ceramide and antiapoptotic S1P determines cell fate.^14^

The intracellular concentration of S1P is regulated in three different ways. First, S1P is released extracellularly by the ATP-independent transporter Spinster 2 (SPNS2) or the ATP-dependent ABC transporter.^15^ Second, S1P is converted into sphingosine by S1P phosphatase (SPP) or irreversibly cleaved by S1P lyase (SPL) into phosphoethanolamine and hexadecenal.^16,17^ Additionally, lipid phosphate phosphatases (LPPs) located in the outer cell membrane regulate extracellular S1P levels.^18^

Platelets, endothelial cells and erythrocytes are the main sources of S1P in plasma, where S1P is bound to high-density lipoprotein (HDL) and albumin.^19,20^ Ito et al. showed that the high concentration of S1P in erythrocytes was caused by a unique lack of S1P lyase and phosphatase expression.^21^ Consistently, increased S1P concentrations are found in blood vessels (micromolar), while other tissues exhibit lower concentrations (nanomolar) due to increased activities of degrading enzymes.^22^

To date, five G-protein coupled S1P receptors have been identified (S1PR1–5). Once activated, downstream signaling pathways lead to altered migration, adhesion, survival, proliferation and angiogenesis.^23^ While S1PR1–3 are expressed in various cell types, S1PR4 and S1PR5 expression is restricted to lymphoid and hematopoietic tissues, as well as the central nervous system and the spleen, respectively.^16,24^ In bone, S1PR 1–3 have been shown to be expressed by both primary OBs and OCs.^25^ The diversity of the S1PR-mediated response to S1P depends on differential coupling to various G-proteins. While S1PR1 is primarily coupled to G_i/o_, S1PR2 and S1PR3 couple to G_12/13_, as well as to G_q_, G_s_ and G_i_.^26^

## Recruitment of osteoclast precursors

As osteoporosis has been shown to be related to increased recruitment of bone marrow-derived osteoclast precursors (OCPs), controlling the migration of cells to the remodeling site was suggested to be a therapeutic option to reverse bone degradation.^27^

**Fig. 2:**
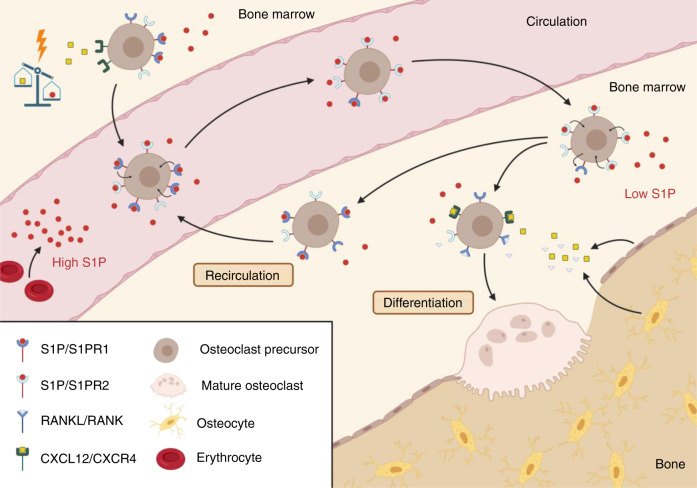
The role of sphingosine-1-phosphate in the recruitment of osteoclast precursors to resorption sites. Under stress conditions, the equilibrium between C-X-C motif chemokine 12 (CXCL12) and sphingosine-1-phosphate (S1P) is shifted in favor of S1P, leading to S1P receptor 1 (S1PR1)-driven chemoattraction of osteoclast precursors toward high S1P levels in the circulation. In a high S1P environment, S1PR1 is internalized, and S1PR2 becomes predominant. Consequently, osteoclast precursors migrate back to the bone marrow through S1PR2-driven chemorepulsion. Activation of S1PR2 decreases S1PR2 expression and increases S1PR1 expression on the cell surface, allowing recirculation into the bloodstream. To prevent recirculation, osteocytes secrete receptor activator of nuclear factor kappa-Β ligand (RANKL) and CXCL12, resulting in the decreased expression of S1PR1 and increased chemoattraction toward bone tissue and enhanced osteoclastogenesis

Although C-X-C motif chemokine 12 (CXCL12) is considered the predominant chemoattractant for bone marrow-derived OCPs, S1P has emerged as another chemoattractant despite comparatively lower potency than CXCL12. Under physiological conditions, comparatively high concentrations of CXCL12 and low concentrations of S1P can be detected in the bone marrow (BM), which is opposite to the corresponding plasma levels. This condition favors the retention of OCPs in the BM, rather than their release into the circulation.^22,28^ Under stress conditions such as OFs, this homeostasis changes (Fig. 2). Therefore, decreased CXCL12 levels and increased S1P levels in the BM lead to increased OCP recruitment to the blood.^22^

To investigate the role of the five S1PRs in OCP recruitment, Ishii et al. showed that S1PR1 and S1PR2 were preferentially expressed in OCPs. Additionally, stimulation with receptor activator of nuclear factor kappa-Β ligand (RANKL) significantly decreased the expression of S1PR1 but not S1PR2. In vitro, OCPs exhibited positive chemotactic responses to S1P, and these effects were attenuated by RANKL stimulation and blocked by pertussis toxin (G_i_ protein inhibitor). Furthermore, S1P increased guanosine triphosphate (GTP)-Rac levels, suggesting that Rac and G_α/I_ are involved in S1PR1-dependent chemotaxis of OCPs. Using intravital two-photon imaging, the authors further demonstrated that OCPs became motile and migrated into blood after the administration of the selective S1PR1 agonist SEW2871 in vivo. Moreover, Ishii et al. generated osteoclast/monocyte-specific S1PR1-deficient (S1PR1^−/−^) mice and showed an osteoporotic phenotype with decreased bone density as a result of increased deposition of S1PR1^−/−^ OCs on the bone surface.^29^

The chemotactic property of OCPs was S1P concentration-dependent, peaked in the nanomolar range and had a sharp decrease in the transition to the micromolar range. Interestingly, blocking the G_i_-coupled signaling downstream of S1PR1 with pertussis toxin leads to diminished migratory potential compared to the absence of S1P, indicating a negative chemotactic or chemorepulsive effect on OCPs. In addition, using S1PR2-targeting RNA interference in OCPs in the presence of high S1P concentrations, the chemorepulsive effect vanishes. To confirm these findings in vivo, S1PR2^−/−^ mice were examined and showed an osteopetrotic phenotype, demonstrating increased bone and trabecular density due to decreased osteoclastic attachment and subsequent resorption. At the cellular level, S1PR2 activates Rho signaling via G_12/13_, which inhibits S1PR1-mediated Rac activity.^30^ Furthermore, Kikuta et al. demonstrated that the osteoanabolic property of the active form of vitamin D (calcitriol) is partly mediated by the suppression of S1PR2 expression in OCPs, leading to the amelioration of bone density in a postmenopausal osteoporotic mouse model.^31^

In summary, S1PR1 is activated by high S1P levels and subsequently becomes internalized, leading to the predominance of S1PR2 on the OCP cell surface within the vasculature and the migration of OCPs into the BM by S1PR2-driven chemorepulsion.^29,30,32^ Conversely, the activation of S1PR2 decreases S1PR2 expression, increases S1PR1 expression, and thereby enables OCPs to recirculate toward the vasculature.^26^ To prevent recirculation, stress-induced apoptotic osteocytes express RANKL and CXCL12, leading to decreased expression of S1PR1, chemoattraction toward the bone tissue, and increased osteoclastogenesis.^29,33,34^

## S1P as an osteoclast–osteoblast coupling factor

Coupling factors are required for the spatial and temporal coordination of bone resorption and bone formation. Although various types of coupling signals and regulatory mechanisms have been identified in recent years, the precise molecular mechanisms of OC-OB communication remain controversial and are not yet fully understood. Originally, it was thought that coupling factors such as transforming growth factor beta are stored within the bone matrix and released into the BMU during bone resorption.^35^ However, the inhibition of bone resorption by the chloride channel inhibitor NS3736, which presumably targets the osteoclastic protein CIC-7, was not accompanied by the inhibition of bone formation. Therefore, the resulting hypothesis was formulated that OCs secrete or present coupling factors independent of their resorptive activity.^36^

**Fig. 3:**
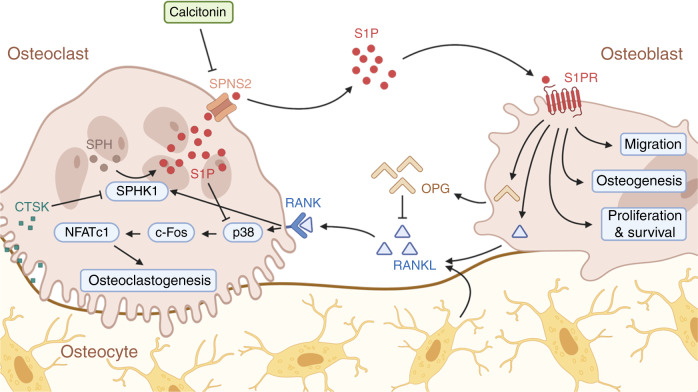
The role of sphingosine-1-phosphate in osteoclast–osteoblast coupling. The binding of receptor activator of nuclear factor kappa-Β ligand (RANKL) to osteoclast precursors leads to osteoclastogenesis, as well as increased sphingosine kinase (SPHK1) expression and activity, resulting in high sphingosine-1-phosphate (S1P) levels. S1P acts in an intracrine manner by inhibiting osteoclastogenesis, and also through paracrine means. By binding to its S1P receptors (S1PR) on osteoblasts, S1P regulates fundamental cellular processes such as migration, osteogenesis, proliferation and survival. Furthermore, S1P affects the ratio of secreted osteoprotegerin (OPG) and RANKL. Intracellular S1P levels in osteoclasts are lowered by a cathepsin K (CTSK)-mediated decrease in SPHK1 expression and are increased by calcitonin-mediated downregulation of Spinster 2 (SPNS2) expression

To identify potential coupling factors, Ryu et al. demonstrated for the first time that the binding of RANKL to bone marrow-derived macrophages (BMMs) leads to osteoclastic differentiation through p38, c-Fos, and NFATc1 (Fig. 3). Additionally, increased SPHK1 expression and activity, which resulted in high S1P levels, were observed during osteoclastic differentiation. Ryu et al. observed that S1P acts as both an intracellular messenger and an extracellular signaling molecule. In a BMM monoculture, intracellular S1P was shown to create a negative feedback loop during osteoclastogenesis by suppressing p38 activity, while extracellular S1P had no effect.^37^

As mentioned previously, S1P can be released from OCs into the BMU via the transporter SPNS2. We previously demonstrated that calcitonin, a hormone that inhibits bone resorption and thereby regulates calcium homeostasis when applied pharmacologically, binds to the calcitonin receptor on murine OCs, leading to decreased expression of *Spns2* and a subsequent increase in intracellular S1P. Calcitonin receptor-deficient mice exhibit increased blood and bone S1P levels, which are accompanied by elevated bone formation rates. Interestingly, similar to that of SPHK1, increased expression of SPNS2 was observed during osteoclastogenesis.^25^ In vitro, adding S1P to BMMs cocultured with OBs led to the potentiation of osteoclastogenesis as a result of enhanced RANKL and decreased osteoprotegerin (OPG) expression in OBs. Moreover, S1P mainly activates p38 and extracellular-signal regulated kinases (ERK) to increase cyclooxygenase-2 (COX2) levels, resulting in increased prostaglandin E_2_ (PGE2) production and subsequently enhancing RANKL expression.^37^

While our study confirmed the S1P-mediated decrease in the OPG/RANKL ratio in murine bone marrow-derived OBs after incubation with 1 μM S1P for 6 h, Matsuzaki et al. were the first to report an increase in the OPG/RANKL ratio in the osteoblast-like cell lines SaOS-2 and MC3T3-E1 after incubation with 1 μM S1P for 8 h.^25,38^ The authors showed S1P-induced activation of the phosphoinositide 3-kinase/protein kinase B (PI3K/AKT) signaling pathway, resulting in glycogen synthase kinase 3β (GSK-3β) inhibition and the promotion of β-catenin nuclear translocation and the subsequent increase in OPG expression.^38^ However, it remains unclear whether the different effects on the OPG/RANKL ratio are due to the different cell lines, their different differentiation stages and associated stage-dependent S1PR expression or different incubation times.

The presence of S1P within the BMU also directly affects OB differentiation and bone formation. *Ctsk*^*−/−*^ mice that lack the lysosomal protease cathepsin K show an osteosclerotic phenotype with increased bone volume and bone formation rates, accompanied by high numbers of OCs and OBs. Conditional knockout of *Ctsk* in murine OCs in vitro leads to increased SPHK1 expression, whereas the presence or activity of cathepsin K in wild-type OCs represses SPHK1 expression, suggesting a potential role in the temporal uncoupling of bone resorption and bone formation. Consistent with this finding, conditioned media from *Ctsk*-deficient OCs contains increased S1P levels. Culturing OBs with conditioned media from *Ctsk*-deficient OCs resulted in increased alkaline phosphatase (AP) activity and mineralization, while the S1PR1/3 antagonist VPC23019 blunted these effects.^39^ Moreover, we verified that the osteoanabolic effect of S1P was mediated by S1PR3 using the structural sphingosine analog fingolimod (FTY720), the daily application of which increased bone formation in WT but not S1PR3-deficient mice.^25^

In addition to increased S1P production and secretion, the S1P concentration within the BMU is also increased by inhibiting its degradation. Weske et al. demonstrated that inactivating S1P degradation by genetic and pharmacological blockade of S1P lyase in vivo leads to an increase in bone mass and bone strength. S1PR2 was identified as a crucial receptor that not only promotes osteoblast differentiation but also inhibits osteoclastogenesis by increasing the OPG/RANKL ratio. Thus, while S1PR2-deficient mice suffer from osteopenia, inhibiting S1P lyase successfully ameliorated osteoporosis caused by genetic ablation of OPG in mice.^40^ Additionally, the S1PR2 agonist CYM5520 corrected ovariectomy-induced osteopenia in mice by increasing bone formation.^41^ Interestingly, although S1PR3 deficiency normalized the skeletal phenotype in calcitonin receptor-deficient mice with high S1P levels, it did not affect the skeletal phenotype of mice lacking S1P lyase, indicating differential functions of S1P receptors depending on the experimental or pathophysiologic context.^25,42^ Likewise, it is possible that *Sglp1* deficiency-induced high extracellular S1P levels cannot be compensated by S1PR knockout alone. Therefore, it would be interesting to repeat the experiment with S1PR2- and S1PR3-deficient mice.^42^

In vitro, S1P was shown to affect OB differentiation and activity in an autocrine manner. Brizuela *et al*. observed increased SPHK1 and S1PR3 expression in MC3T3 cells during differentiation. Pharmacological inhibition of SPHK1, an S1P-neutralizing antibody, or antagonizing S1PR3 with VPC23019 reduced AP activity and the expression of RUNX2, a key transcription factor in OB differentiation, while antagonizing S1PR1 and S1PR2 with W146 and JTE013, respectively, showed no effect. Therefore, Brizuela et al. suggested an S1PR3-mediated autocrine effect of S1P on OBs.^43^

Additionally, OC-derived S1P was shown to stimulate mesenchymal stem cell chemokinesis (random cell movement) in vitro by activating the JAK/STAT3 and FAK/PI3K/AKT signaling pathways via S1PR1 and S1PR2, respectively.^44,45^ In OB precursors, the chemokine platelet-derived growth factor (PDGF) is secreted by OC during bone remodeling and mediates chemotaxis to the resorption site.^46^ Roelofsen et al. demonstrated that the effect of chemotaxis toward PDGF was inhibited by adding S1P to MC3T3-E1 OB precursors in vitro. After bone morphogenetic protein 2-induced differentiation, MC3T3-E1 OBs expressed less S1PR2 and became relatively insensitive to S1P-mediated chemotactic inhibition toward PDGF, suggesting that S1PR2 was a chemorepellent receptor. In addition, S1P-mediated chemorepulsive effects on OB precursors were abolished using S1PR2 targeting RNA interference and the S1PR2 antagonist JTE-013, suggesting that the recruitment of OBs to the resorption site was regulated by a delicate balance between PDGF, S1P and cell differentiation-dependent S1PR2 expression.^47^

Various in vitro studies revealed that S1P controls bone formation by regulating OB proliferation and survival.^48–52^ Dziak et al. were one of the first to demonstrate that short-term incubation with S1P leads enhances proliferation rate in primary human osteoblastic cells, as well as in the human osteosarcoma cell lines MG63 and G292, in a pertussis toxin-sensitive manner. Therefore, the researchers suggested the involvement of S1PR1.^49^ This finding was confirmed by the work of Tantikanlayaporn et al., who used the S1PR1 agonist SEW2871 to increase the proliferation rates of human OBs (CC-2538). Intriguingly, application of SKi, a specific SPHK1 inhibitor, decreased the estradiol-induced proliferative effect, suggesting an overlap in S1P and estradiol signaling.^52^ Additionally, Grey et al. showed that S1P alleviated the apoptosis rates of primary rat osteoblasts and human osteoblasts (SaOS-2) in vitro via G_i_ proteins and downstream PI3K signaling, while the mitogenic effects required p42/44 mitogen-activated protein kinase activation.^48,50^ In this context, it is well known that long-term treatment with glucocorticoids, including dexamethasone, can lead to osteoporosis due to increased OB apoptosis. Ji et al. demonstrated that stimulation with K6PC-5, a novel, specific and direct activator of SPHK1, and the subsequent increased S1P levels attenuated dexamethasone-induced apoptosis in cultured osteoblasts (MC3T3-E1 and primary murine osteoblasts).^51^

Based on its dual modes of action on both bone-forming osteoblasts and bone-resorbing osteoclasts, most studies investigating the effects of S1P specifically focus on trabecular bone remodeling. In the case of periosteal bone formation and cortical bone, however, only limited data are available. Lotinun et al. found that the increased bone formation in mice with osteoclast-specific deletion of cathepsin K not only resulted in elevated trabecular bone mass but also an increase in total cross-sectional volume and cortical bone volume without any change in marrow volume, suggesting that S1P-dependent osteoclast-osteoblast coupling may indirectly affect periosteal bone formation.^39^ Likewise, the coupling of angiogenesis with bone formation in the periosteal environment was shown to be dependent on platelet-derived growth factor-BB-induced Akt/FAK-dependent S1P signaling, which was initiated by preosteoclasts residing on the periosteal surface of cortical bone.^53^ In terms of cortical bone, mice with elevated S1P levels due to S1P lyase-deficiency showed a S1PR3-independent decrease in cortical bone mass and higher cortical porosity, indicative of increased bone resorption.^42^ In contrast, however, S1PR2-deficient mice were reported to exhibit decreased cortical thickness, while the opposite effect was observed in response to pharmacologic inhibition of S1P lyase.^40^ Therefore, although the biological actions of S1P signaling in trabecular bone are comparatively well characterized, the current understanding of its impact on cortical bone is less clear and warrants further mechanistic studies.

## S1P as a biomarker of osteoporosis

For clinicians treating patients with osteoporosis, it is important to determine fracture risk and monitor the efficacy of antiresorptive or osteoanabolic therapy. To date, bone mineral density (BMD) is assessed by dual-energy X-ray absorptiometry and is the gold standard for diagnosing osteoporosis. Because a large proportion of OFs occur in patients with T-scores above −2.5, the Fracture Risk Assessment Tool (FRAX) was developed by the World Health Organization to improve the prognostic sensitivity of BMD. The computer-based algorithm ascertains the risk of an OF within the next 10 years by combining the measured BMD with clinical risk factors, including age, sex, smoking status and glucocorticoid therapy.^54^

Bone strength is the result of both high bone density and good bone quality. However, in clinical practice, only bone density and not quality is determined, as there are no suitable methods to quantify bone quality yet. In recent years, bone turnover markers in blood and urine have been established to provide insight into bone metabolism.^55^ These markers can be classified as bone formation markers (BFMs), such as the hormone osteocalcin, bone AP and procollagen type I N-terminal propeptide (PINP), and bone resorption markers (BRMs), such as C- and N-terminal telopeptides of type I collagen (CTX and NTX).^55^ To assess the relative balance between bone resorption and bone formation, Eastell et al. calculated the uncoupling index by subtracting an age-adjusted *Z* score for BRMs from an age-adjusted *Z* score for BFMs. Therefore, a positive uncoupling index would indicate that bone remodeling was unbalanced in favor of bone formation.^56^ As discussed previously, many in vitro and in vivo experiments have demonstrated the role of S1P in the recruitment of osteoclasts to the resorption site, as well as in osteoclast-osteoblast coupling. Therefore, various studies have increasingly focused on blood S1P levels as a potential biomarker for osteoporosis. S1P levels were assayed using a commercial S1P competitive ELISA kit or by liquid chromatography-tandem mass spectrometry (for a complete list, please see Table 1).

**Table 1:** Role of sphingosine-1-phosphate levels as biomarkers for osteoporosis

Reference	Study		S1P Source	Correlation/Association	
	Type	Population		Positive	Negative
^65^	cross-sectional	patients undergoing hip replacement surgery (*n* = 16)	BM		S1P ↔ prevalence of hip fracture
^59^	prospective cohort	postmenopausal women (*n* = 707)	Plasma	S1P ↔ OF risk (independent of BMD) S1P ↔ BRM	S1P ↔ BMD
^57^	longitudinal cohort	postmenopausal women (*n* = 248)	Plasma	S1P ↔ prevalence & incidence of OF (independent of BMD and BRM) S1P ↔ BRM S1P ↔ insufficient response to bisphosphonate therapy	
^58^	case–control	postmenopausal women with or without VF (*n* = 138)	Plasma	S1P ↔ prevalence of VF (independent of BMD) S1P ↔ BRM	S1P ↔ BMD
^66^	case–control	patients undergoing hip surgery (*n* = 70)	Plasma & BM	S1P Plasma/BM ratio ↔ prevalence of osteoporotic hip fracture	
^62^	cross-sectional	postmenopausal women (*n* = 357)	Plasma	S1P ↔ BRM S1P ↔ serum calcium	S1P ↔ BMD (femur) S1P ↔ uncoupling index
^60^	cross-sectional case–control	postmenopausal women and men with or without OF (*n* = 422)	Plasma	S1P ↔ OF risk (independent of FRAX)	
^63^	cross-sectional	postmenopausal women (*n* = 339)	Plasma		S1P ↔ TBS S1P ↔ BMD (femur neck)
^80^	cross-sectional	postmenopausal women and men (*n* = 131)	Serum		
^61^	case–control	postmenopausal women with or without VF (*n* = 116)	Plasma	S1P ↔ prevalence of OF	
^40^	cross-sectional	women and men (*n* = 4 091)	Serum	S1P ↔ BFM S1P ↔ serum calcium	S1P ↔ PTH S1P ↔ QUS based bone stiffness

*BM* bone marrow, *BMD* bone mass density, *BFM* bone formation marker, *BRM* bone resorption marker, *FRAX* fracture risk assessment tool, *OF* osteoporosis-related fracture, *PTH* parathyroid hormone, *S1P* sphingosine-1-phosphate, *TBS* trabecular bone score, *VF* vertebral fracture, *QUS* quantitative ultrasound-based

Most studies published to date have consistently shown that high plasma S1P levels are associated with an increased prevalence and incidence of OFs^57,58^ and that S1P levels positively correlate with the risk for OFs.^59–61^ In two studies, the statistical significance even persisted when the data were adjusted for BMD or FRAX, suggesting that plasma S1P is an independent risk factor for OFs.^59,60^ In terms of bone remodeling markers, the majority of available studies also showed a positive correlation between plasma S1P levels and BRMs.^57–59,62^ By determining the uncoupling index of study participants, Lee et al. showed a negative correlation with S1P levels, and it was concluded that increased circulating S1P concentrations correlated with unbalanced bone remodeling in favor of bone resorption.^62^ These effects appear to be independent of parathyroid hormone (PTH), as a positive correlation was found between S1P and serum calcium, and a negative correlation was found between S1P and PTH.^40,62^ In terms of bone mass, most studies reported negative correlations between S1P levels and BMD.^58,59,62,63^ In addition, Lee et al. described an inverse correlation with the trabecular bone score, which is a surrogate marker for the quality of trabecular microarchitecture.^63,64^ In sharp contrast, a large investigation by Weske et al. included 4091 participants and reported a positive association between S1P and the BFM PINP and a negative association with the QUS-based bone stiffness index.^40^ Based on the lack of a negative association between S1P and CTX, the authors concluded that S1P is a driver of bone formation and may serve as a counterregulatory measure to increase bone mass in response to decreasing bone quality.

Considering these conflicting findings, assessments of plasma S1P levels alone must be interpreted carefully, as S1P gradients are not taken into consideration, and the available assays may be influenced by the relative abundance of S1P-carrier proteins. In this context, Ahn et al. were among the first to measure bone marrow (BM) S1P levels in 16 patients who underwent hip replacement surgeries and found significantly lower bone BM S1P levels in patients with hip fractures.^65^ Subsequently, Kim et al. reported a positive correlation between the plasma/BM S1P ratio and the risk for osteoporotic hip fractures, emphasizing the potential relevance of assessing S1P gradients in musculoskeletal disease.^66^ Regarding the reliability of plasma S1P measurements, Song et al. observed that the positive correlation between total plasma S1P and the risk of OFs was independent of the concentrations of S1P-carrier proteins, including HDL, LDL, and albumin. If confirmed in future studies, this finding could simplify the assessment of S1P in clinical routines and make this process less expensive.^61^

Collectively, most findings have provided clinical evidence that systemic S1P and/or the S1P plasma/BM ratio may serve as a biological marker to predict the risk of OFs, while the largest study thus far by Weske et al. showed that S1P was positively associated with bone formation. Therefore, further large-scale studies with additional ethnic groups are required, especially because most clinical reports seem to contradict the skeletal effects of S1P observed in animal models. These discrepancies might be related to the fact that local S1P-dependent signaling events within the BMU may not necessarily reflect the systemic situation, since S1P concentrations in the blood are much higher than those in other tissues, including bone.^67^ Alternatively, it has been suggested that the primary effect of S1P on human bone metabolism involves the recruitment of OCPs rather than an osteoanabolic effect within the BMU.^57–59^ In this context, high S1P levels would result in the internalization of S1PR1 and a predominance of S1PR2 on the cell surface of OCPs, subsequently augmenting the migration of OCPs into the BM by S1PR2-driven chemorepulsion.^30^ Likewise, the bone-protective effect of fingolimod, a selective agonist of S1PR1/3 but not S1PR2 that is used to treat multiple sclerosis, may be attributed to reducing the number of mature osteoclasts on the bone surface by promoting the S1PR1-driven recirculation of OCPs.^29,68^ The fact that the association of S1P levels with OFs was partly independent of BMD supports the notion that the net impact of circulating S1P in humans may be exerted through impaired cellular bone remodeling rather than a reduction in bone mass.^40,57–59^ These considerations warrant further mechanistic studies that take into account the complex regulation of S1P receptor signaling, which depends on differential S1P gradients.

## S1P and S1PRs as potential targets for the treatment of osteoporosis

To date, two different groups of drugs are used to treat osteoporosis: antiresorptive drugs and osteoanabolic drugs.^69^ Antiresorptive drugs, including bisphosphonates or denosumab, a monoclonal antibody that neutralizes RANKL, inhibit osteoclastic bone resorption. However, in addition to potentially severe adverse effects, these drugs also inhibit OC-OB coupling and concomitantly impact de novo bone formation.^69–71^ Osteoanabolic drugs, including intermittent parathyroid hormone (iPTH) administration and romosozumab, stimulate osteoblastic bone formation. Romosozumab, a recently approved monoclonal antibody against sclerostin, disinhibits the osteoanabolic Wnt signaling pathway and leads to an increase in OB numbers and activity but also attenuates physiological OC-OB coupling.^72^

As currently available treatment options for patients with osteoporosis remain comparatively limited, targeting S1P and its receptors could represent a new approach for osteoporosis therapy. This is especially interesting, as S1P signaling not only affects OCP recruitment but also acts on OC-OB coupling to promote bone formation, contrary to currently available drugs. Therefore, targeting S1P signaling may restore the dynamic equilibrium within the bone by avoiding excessive inhibition or activation of OCs and OBs, respectively. However, the various mechanisms of action of S1P in bone metabolism are highly complex and are regulated by the dynamic expression of S1PR and S1P transporters on cell surfaces, as well as by the synthesis and degradation of S1P. Historically, the first approved drug that targeted S1P signaling was the structural sphingosine analog fingolimod. The phosphorylated, active form of fingolimod is a functional agonist for S1PR1 on lymphocytes, and its binding leads to the internalization of S1PR, thereby inhibiting S1PR1-dependent egress of lymphocytes from secondary lymphoid organs. As a result, fewer autoreactive lymphocytes can cross the blood–brain barrier, and disease relapse in multiple sclerosis is attenuated.^73^

According to the results of Ishii et al., S1PR1 and S1PR2 exhibited opposing effects on the recruitment of OCPs to the resorption site. While systemic application of the S1PR1 agonist SEW2871 decreased the number of OCs on the bone surface by increasing their rate of recirculation into the bloodstream, the S1PR2 antagonist JTE013 reduced OCP migration from the bloodstream into the BM by preventing S1PR2-driven chemorepulsion in mice.^29,30^ In addition, a systemically administered S1PR2 antagonist enhanced murine OB precursor recruitment to the resorption site by disinhibiting chemotaxis towards PDGF, while an S1PR1 agonist increased the proliferation rates of human OBs.^47,52^ In contrast, an S1PR2 antagonist attenuated OB differentiation and disinhibited osteoclastogenesis by downregulating the OPG/RANKL ratio, whereas a S1PR2 agonist increased OB numbers and activity in mice.^40,41^ Therefore, it is essential to distinguish between systemic and local application of S1PR agonists and antagonists. A further therapeutic option could be to use a selective S1PR3 agonist, as our group previously demonstrated that S1PR3 agonism promoted murine bone formation in vivo.^25^ Moreover, Brizuela et al. showed an S1PR3-dependent stimulatory effect on human OB differentiation in vitro.^43^ However, it is important to note that although a local, bone-specific approach to S1PR3 agonism may be beneficial for bone remodeling, systemic administration could induce adverse drug reactions such as bradycardia due to S1PR3 expression in pacemaker cells in the cardiac atrium.^74^

Because, in addition to specific S1PR agonists and antagonists, fingolimod also exerted beneficial effects in the treatment of osteoporosis, the enzymes involved in S1P synthesis and degradation have received considerable attention.^68^ Both the activation of SPHK and inhibition of S1P lyase increase S1P levels. In a model of glucocorticoid-associated osteoporosis in vitro, Ji et al. showed that the activation of SPHK1 in murine OBs using K6PC-5 increased S1P production and that the subsequent enhanced phosphorylation of AKT protected against glucocorticoid-induced cell death.^51^ Moreover, Weske et al. reported that inhibiting S1P lyase with 4-deoxypyrodoxin and the subsequent increase in S1P concentration enhanced bone formation, as well as bone strength and bone mass in mice, accompanied by reduced OC activity as a result of an increase in the OPG/RANKL ratio. Compared to those of osteoanabolic iPTH therapy, similar potent osteoanabolic effects of S1P lyase inhibition were observed in an ovariectomy-induced osteoporosis mouse model by Weske et al. However, as iPTH shows no simultaneous antiresorptive effect and should be discontinued after 2 years due to a subsequent increase in osteoclast activity, targeting S1P lyase may represent an elegant approach due to its simultaneous antiresorptive and osteoanabolic effects.^40^ As a future perspective, it would be interesting to study the impact of SPNS2 inhibitors on osteoclast formation, as this transporter protein regulates intracellular S1P levels and may effectively control osteoclastogenesis. To the best of our knowledge, there have been no published studies to date, but a patent for components of such an inhibitor has been registered.^75^

In light of these possibilities, it is also noteworthy that targeting S1P metabolism to treat bone disease may cause nonspecific or undesired side effects. For example, systemic administration of fingolimod alone does not improve fracture healing in a mouse model, and increased S1P levels due to S1P lyase inhibition can promote S1PR1-mediated immunosuppression in mice.^41,76^ Thus, it can be deduced that tissue- or cell-specific targeting of S1P-related drugs will be increasingly important to translate the discoveries regarding S1P metabolism into broader clinical applications. One possible way to decrease the off-target effects of S1P-related drugs and increase pharmacologic drug levels in bone tissue could be through conjugation to bisphosphonates. For example, Doschak et al. examined a rat model of osteoarthritis and showed that conjugating OPG to bisphosphonates resulted in twofold higher concentrations in bone than unmodified OPG, thereby increasing the OPG/RANKL ratio.^77^ However, it is important to note that bisphosphonates have low oral bioavailability and cause gastrointestinal irritation, such as nausea and pain when swallowing, explaining the prevalence of low therapy adherence.^78^ Another possibility lies in the development of bispecific antibodies. Chen et al. could control the distribution of denosumab to murine bone tissue by fusing denosumab with single chain variable fragments of an antibody that was specific for osteonectin, which is amply expressed in osseous tissues.^79^ Therefore, a bispecific antibody that binds to a bone-specific antigen and neutralizes S1P lyase may prove useful in promoting bone formation and simultaneously limiting bone resorption.

## Concluding remarks

In conclusion, through great efforts in basic, translational, and clinical research in recent years, it has become clear that S1P metabolism is important in the regulation of bone remodeling. S1P has not only been shown to regulate bone resorption and bone formation, thus controlling bone mass accrual and bone quality, but may also serve as a novel biomarker to predict osteoporosis-related fractures. Targeting S1P receptors or the enzymes involved in S1P turnover has revealed promising approaches for the generation of novel treatment options for patients with osteoporosis. However, based on the delicate and tightly controlled equilibrium of S1P signaling, which involves the generation and degradation of S1P, as well as the differential expression of S1P receptors by a multitude of different cell types, further research is necessary to translate these findings into clinical practice.
